# Assessing the Impact of the COVID-19 Pandemic on the Severity of Pediatric Inflammatory Bowel Disease Admissions and New Diagnoses

**DOI:** 10.1093/crocol/otad062

**Published:** 2023-10-25

**Authors:** Malika Waschmann, Ariana Stuart, Kimberly Trieschmann, Henry C Lin, Anna K Hunter

**Affiliations:** Department of Pediatrics, University of Washington, Seattle Children’s Hospital, Seattle, WA, USA; School of Medicine, Oregon Health & Science University, Portland, OR, USA; Department of Pediatrics, University of Washington, Seattle Children’s Hospital, Seattle, WA, USA; Division of Pediatric Gastroenterology, Doernbecher Children’s Hospital, Portland, OR, USA; Department of Pediatrics, Oregon Health & Science University, Portland, OR, USA; Division of Pediatric Gastroenterology, Doernbecher Children’s Hospital, Portland, OR, USA; Department of Pediatrics, Oregon Health & Science University, Portland, OR, USA; Division of Pediatric Gastroenterology, Doernbecher Children’s Hospital, Portland, OR, USA; Department of Pediatrics, Oregon Health & Science University, Portland, OR, USA

**Keywords:** pediatric IBD, VEO-IBD, COVID-19, disease severity, telehealth

## Abstract

**Introduction:**

The COVID-19 pandemic has introduced new challenges to the diagnosis and management of pediatric inflammatory bowel disease (IBD). Many patients have had only limited access to their providers through telemedicine, and many chose to delay nonemergent treatment.

**Methods:**

A retrospective chart review of patients with IBD seen by the Pediatric Gastroenterology Division at Doernbecher Children’s Hospital from January 2018 to August 2021 was conducted. The study cohort was divided into 2 groups: those presenting before the onset of the COVID-19 pandemic (January 1, 2018 to February 28, 2020) and those presenting during the pandemic (March 1, 2020 to August 1, 2021). Variables collected included: age, sex, race, ethnicity, IBD type, insurance type, location of residence. Primary outcome measures selected focused on disease severity, initial type of treatment, or surgical intervention offered. A subgroup analysis of the new diagnosis patients was performed. Data were analyzed using independent *t*-tests, chi-squared analysis, and Wilcoxon rank sum tests.

**Results:**

Two hundred and eleven patients met inclusion criteria, 107 (72 new diagnoses, 35 admissions) within the pre-COVID epoch and 104 (67 new diagnoses, 37 admissions) within the during-COVID epoch. Patients in the during-COVID epoch had higher fecal calprotectin level and were more likely to be started on a biologic as initial treatment. Patients admitted during COVID for IBD flare were more likely to require surgical intervention. Subgroup analysis of newly diagnosed patients revealed higher incidence of comorbid depression and anxiety.

**Conclusions:**

Our review identified increased disease severity in newly diagnosed pediatric patients with IBD as well as pediatric patients admitted for flare during COVID. Increases in anxiety and depression rates during COVID may have contributed to worsened disease severity.

## Introduction

Inflammatory bowel disease (IBD) encompasses a spectrum of chronic autoimmune disorders of the gastrointestinal tract that includes Crohn’s disease (CD), ulcerative colitis (UC), indeterminate colitis (IC), and very early onset IBD (VEO-IBD). Twenty to thirty percent of cases of IBD are diagnosed in pediatric patients with a prevalence of 77 per 100 000 children.^1,2^ The current incidence of pediatric IBD in Northern America is about 2.4 to 15.4 per 100 000 person-years. Trends around the world are demonstrating that the incidence of pediatric IBD across all ages is generally increasing, with CD maintaining as more common than UC.^3–6^ There were an estimated 119 282 hospitalizations of pediatric patients with IBD in the United States between 2002 and 2015.^7^

The etiology of IBD is multifactorial including immunologic, genetic, and environmental contributors. The hygiene hypothesis states that immune system development—specifically establishment of the microbiome—is in part determined by childhood exposure to environmental antigens. More diverse exposures allow for more robust development, and vice versa.^8^ Through this lens, autoimmunity may in part be attributed to a lack of heterogeneity of environmental exposures in childhood, a situation that becomes much more common as robust sanitation measures are employed in urban centers.^9,10^ The modern, sanitation-reliant approach to hygiene has substantially increased since the onset of the COVID-19 pandemic, which not only encouraged increased use of sanitizing products, but also resulted in mass environmental isolation. While this approach may have proved beneficial in decreasing spread of the SARS-CoV-2 virus, the hygiene hypothesis suggests it may have long-term impact on the incidence of autoimmune diseases such as IBD.^8^

The relapsing and remitting course of IBD suggests a role of both internal and external triggers for worsened disease severity. Both anxiety and depression as well as general stress can influence our gut through a variety of mechanisms. Physical and/or emotional stress leads to corticotrophin-releasing factor (CRF) release and downstream cortisol production.^11^ CRF has been implicated in changes in gastrointestinal motility.^12^ Furthermore, CRF contributes to increased mucosal permeability by triggering mast cell degranulation and TNF-a release.^13^ Stress modulates additional cytokine release via sympathetic nervous system activation and release of catecholamines^14^ mediating mucosal inflammation and permeability through activation of nuclear factor κB signaling and vagal nerve inhibition.^15,16^

Bidirectional effect of depression on inflammation has also been studied with worsened inflammation shown by elevation of proinflammatory cytokines (IL-1, IL-6, IL-12, and TNF-a) described in depressed individuals.^17^ It is through these mechanisms among others, that emotional and physical stress effect IBD onset, course, and prognosis. For example, academic stress at the start of new semesters sees a rise in IBD symptom onset in pediatric patients.^18^ Rates of both depression and anxiety symptoms correlate with relapse and worsening of disease by both perceived symptom severity and clinical endpoints. Lastly, overall prognosis as marked by rates of surgical intervention and hospitalization is worse in patients with underlying mood disorders.^19,20^

The COVID-19 pandemic has contributed to a multitude of stresses faced by children and their families. The impact of the COVID-19 pandemic on the pediatric healthcare landscape is marked by changes in both the quantity and type of general pediatric admissions and pediatric admissions specifically for IBD in the United States. Children’s hospitals reported a reduction in general pediatric inpatient admissions of 45.4% during April of 2020 compared to the years prior, with similar findings in other countries including Australia and the Netherlands.^21,22^ The hypotheses behind these reductions are multifactorial. Social distancing and masking mandates likely contributed to reductions in admissions for acute respiratory failure given most cases are due to infectious etiologies.^23–25^ Reductions in opportunities for in-person sports and school activities may have contributed to reductions in trauma admissions.^26^ Parental surveys have also suggested that fears regarding exposure to COVID-19 underlie reductions in admission rates.^27^ Analyses of admission data and endoscopies for pediatric patients with IBD have noted similar reductions in admissions. A multicenter study in Italy noted reductions in IBD-specific hospitalizations during the COVID-19 pandemic of greater than 50%, most notably for newly diagnosed patients. Admission for relapses and surgical interventions remained consistent with pre-COVID rates.^28^ Thus far, no prior studies have compared the severity of newly diagnosed pediatric IBD patients or the severity of IBD flares prior to and during the COVID-19 pandemic.

The objective of this study was to assess the impact of the COVID-19 pandemic on the pediatric IBD population receiving care at Doernbecher Children’s Hospital. Specifically, this study sought to investigate any effect on both the incidence of new diagnoses or admissions and severity of disease status during those interactions with the healthcare system.

## Methods

A retrospective chart review was conducted on patients seen by the Pediatric Gastroenterology Division at Doernbecher Children’s Hospital at OHSU with IBD from January 2018 to August 2021. Patients were included in this review if they were ≤18 years of age and either newly diagnosed with IBD or had a known diagnosis of IBD and were admitted for management of an IBD flare. The study cohort was divided into 2 groups: those presenting before the onset of the COVID-19 pandemic (January 1, 2018 to February 28, 2020) and those presenting during the pandemic (March 1, 2020 to August 1, 2021). For patients who met inclusion criteria for both time periods, we prioritized their new diagnosis data. For example, if a patient was newly diagnosed prior to the COVID-19 pandemic and then admitted during the pandemic, we only included their new diagnosis data. For patients who had multiple presentations for admission pre and during COVID we only included the data from their first admission.

Variables collected for all study participants included: classification as a new diagnosis or admission for flare, age, sex, race, ethnicity, IBD type (CD, UC, IC, VEO-IBD), insurance type, location of residence. Primary outcome measures assessing severity of disease included the following: laboratory markers of hemoglobin, albumin, C-reactive protein (CRP), sedimentation rate (ESR), and fecal calprotectin, clinical scoring of disease severity with Physician Global Assessment tool (Part of Improve Care Now EMR smartform), and endoscopic severity scores (Mayo score for UC, IC, and VEO-IBD; and Simple Endoscopic Score [SES] for CD).^29,30^ The initial type of treatment or surgical intervention offered was also included.

A subgroup analysis of the new diagnosis patients was performed. Variables collected included: time from primary care physician referral to initial pediatric gastroenterology (GI) visit, duration of symptoms prior to initial GI visit, type of initial GI visit (virtual or in-person), time from initial GI visit to diagnosis, presence of comorbid atopic or autoimmune diseases, and presence of comorbid psychiatric diseases. Patient access data including the number of patient telephone calls, patient email messages, GI visits, emergency department (ED) visits, and admissions related to IBD were recorded in the 3 months following diagnosis.

Demographic data were analyzed using independent *t*-tests and chi-squared analysis. Lab values were analyzed using independent *t*-tests. Endoscopy scores were analyzed using Wilcoxon rank sum tests. All statistical analyses were performed using SPSS. The study was approved by the Oregon Health & Science University Institutional Review Board.

## Results

This retrospective chart review identified 211 pediatric patients who were newly diagnosed with IBD or admitted for IBD flare during the study time frame, 107 (72 new diagnoses, 35 admissions) within the pre-COVID epoch and 104 (67 new diagnoses, 37 admissions) within the during-COVID epoch. Patient demographic is summarized in Table 1. Patients from both epochs were primarily White and privately insured with a diagnosis of CD. Patient demographic differences between the pre-COVID and during-COVID epoch include ethnicity with 92.4% of patients identifying as non-Hispanic in the during-COVID epoch compared to 82.2% in the pre-COVID epoch (*P* = .03), and area of residence with 64.4% urban in the during-COVID epoch versus 49.5% urban in the pre-COVID epoch (*P* = .03).

**Table 1.** Patient demographics.

		Pre-COVID (*N* = 107)	During-COVID (*N* = 104)	*P*-value
Encounter type^a^	New diagnosis	67.3% (72)	64.4% (67)	.19
	Admission for flare	32.7% (35)	35.6% (37)	
Diagnosis^a^	Crohn’s	58.9% (63)	67.3% (70)	n/a^+^
	UC	33.6% (36)	24.0% (25)	
	IC	3.7% (4)	3.8% (4)	
	VEO-IBD	3.7% (4)	4.8% (5)	
Age (in years)^b^		12.6 (3.5)	13.4 (3.5)	.04*
Gender^a^	Female	47.7% (51)	49.0% (51)	.84
	Male	52.3% (56)	51.0% (53)	
Race^a^	White	92.5% (99)	91.8% (89)	n/a^+^
	Black	1.9% (2)	3.1% (3)	
	AI/AN	0.9% (1)	1.0% (1)	
	Asian	3.7% (4)	2.1% (2)	
	Native Hawaiian	0.9% (1)	2.1% (2)	
Ethnicity^a^	Non-Hispanic	82.2% (88)	92.4% (85)	.03*
	Hispanic	17.8% (19)	7.6% (7)	
Insurance^a^	Private	59.8% (64)	62.5% (65)	.53
	Public	40.2% (43)	36.5% (38)	
Area of residence^a^	Urban	49.5% (53)	64.4% (67)	.03*
	Rural	50.5% (54)	35.6% (37)	

Abbreviations: IC, indeterminate colitis; UC, ulcerative colitis; VEO-IBD, very early onset inflammatory bowel disease.

^a^Statistical analysis for this variable performed with chi-squared test.

^b^Statistical analysis for this variable performed with independent *t*-test.

^+^Unable to calculate chi-square given *n*.

*Denotes statistical significance at α ≤ .05.

There was no difference in presenting disease severity by disease activity score between patients in the 2 study time periods. While endoscopic disease severity as measured by Mayo and SES scores revealed generally higher average scores during-COVID epoch, these differences were not statistically significant (Table 2). However, there were significant difference in initial laboratory findings between patients in the 2 study time periods (Table 3). Patients in the during-COVID epoch were noted to have a lower average hemoglobin, albumin, and higher fecal calprotectin and CRP on initial labs with only over fecal calprotectin difference reaching statistical significance (867.1 mg/kg vs 1653.5 mg/kg, *P* = .001). On subgroup analysis by type of IBD, patients with CD had higher average fecal calprotectin levels on presentation in the during-COVID epoch (CD: 650.5 mg/kg vs 1692.5 mg/kg, *P* = .003). VEO-IBD patients in the during-COVID epoch had a lower average albumin on presentation (4.4 g/dL vs 3.8 g/dL, *P* = .05).

**Table 2.** Initial disease severity and treatment.

		Pre-COVID (*N* = 107)	During-COVID (*N* = 104)	*P*-value
Initial disease activity score^b^	CD	23.1 (14.7)	30.4 (13.3)	.09
	UC	36.7 (13.3)	35.6 (19.9)	.45
	IC	38.3 (16.1)	10.0	.13
	VEO-IBD	no scores recorded	38 (32)	
Endoscopic severity score^b^	Mayo	1.8 (0.9)	2.0 (0.7)	.22
	SES	8.5 (4.1)	11.4 (15.1)	.20
Initial treatment offered following diagnosis^a^	Aminosalicylates	42.9% (42)	31.2% (29)	.02*
	Biologics	20.4% (20)	45.2% (42)	
	6-MP, MTX, Aza	12.2% (12)	6.5% (6)	
	Oral Steroids	13.3% (13)	6.5% (6)	
	Specific Carbohydrate Diet	3.1% (3)	1.1% (1)	
	Antibiotics	2.0% (2)	2.2% (2)	
	Enteral nutrition	5.1% (5)	2.2% (2)	
	Colectomy	1.0% (1)	2.2% (2)	
Surgical intervention^a^	None	99% (106)	93% (97)	.04*
	Colectomy	0% (0)	6% (6)	
	I&D/seton placement	1% (1)	1% (1)	

Abbreviations: 6-MP, 6-mercaptopurine; IC, indeterminate colitis; MTX, Methotrexate; SES, Simple Endoscopic Score; UC, ulcerative colitis; VEO-IBD, very early onset inflammatory bowel disease.

^a^Statistical analysis for this variable performed with chi-squared test.

^b^Statistical analysis for this variable performed with independent *t*-test.

*Denotes statistical significance at α ≤ .05.

**Table 3.** Initial laboratory data.

		Pre-COVID (*N* = 107)	During-COVID (*N* = 104)	*P*-value
Fecal calprotectin^b^	Overall	867.1 (682.8)	1653.5 (1677.4)	.001*
	- Crohns	650.5 (507.3)	1692.5 (1854.7)	.003*
	- UC	1153.9 (847.9)	1691.8 (1338.4)	.09
	- IC	1214.7 (17.4)	882.0 (18.4)	.001*
	- VEO-IBD	1055.3 (867.7)	1447.8 (1417.8)	.35
Hemoglobin^b^	Overall	11.5 (1.8)	11.1 (2.3)	.13
	- Crohns	11.5 (1.5)	11.1 (2.4)	.15
	- UC	11.5 (2.2)	11.3 (2.3)	.37
	- IC	11.8 (2.7)	10.8 (1.7)	.29
	- VEO-IBD	10.4 (0.9)	11.1 (1.4)	.20
Albumin^b^	Overall	3.5 (0.8)	3.3 (0.8)	.08
	- Crohns	3.4 (0.7)	3.1 (0.9)	.06
	- UC	3.5 (0.9)	3.6 (0.7)	.31
	- IC	3.8 (0.9)	3.6 (0.5)	.39
	- VEO-IBD	4.4 (0.4)	3.8 (0.6)	.05*
CRP^b^	Overall	32.6 (40.8)	44.0 (96.2)	.16
	- Crohns	41.2 (44.1)	55.3 (113.8)	.21
	- UC	30.4 (55.9)	22.9 (38.7)	.30
	- IC	17.7 (27.9)	29.2 (36.0)	.32
	- VEO-IBD	3.7 (4.1)	5.4 (4.1)	.29
ESR^b^	Overall	37.2 (28.9)	36.0 (32.0)	.40
	- Crohn’s	41.8 (30.6)	39.2 (32.4)	.33
	- UC	32.3 (25.0)	32.4 (34.4)	.50
	- IC	35.3 (41.2)	23.0 (4.0)	.32
	- VEO-IBD	18.3 (8.3)	17.8 (16.1)	.48

Abbreviations: CRP, C-reactive protein; ESR, sedimentation rate; IC, indeterminate colitis; UC, ulcerative colitis; VEO-IBD, very early onset inflammatory bowel disease.

^b^Statistical analysis for this variable performed with independent *t*-test.

*Denotes statistical significance at α ≤ .05.

There were significant differences in initial treatment regimen between the 2 study time periods (Table 2). Patients in the during-COVID epoch were more likely to be started on a biologic medication as initial treatment (45.2% vs 20.4%, *P* = .02) and less likely to be started on a Aminosalicylates (31.2% vs 42.9%, *P* = .02) or immunomodulator such as Azathioprine, 6-mercaptopurine (6-MP), or Methotrexate (6.5% vs 12.2%, *P* = .02). Furthermore, patients admitted during COVID for IBD flare were more likely to require surgical intervention, particularly colectomy (0% vs 6%, *P* = .04).

Subgroup analysis of newly diagnosed patients in each time period revealed significant differences in comorbid mental health diagnoses (Table 4). Depression (0% vs 10.8%, *P* = .01) and anxiety (4.2% vs 13.8%, *P* = .01) were more common in the during-COVID epoch compared to the pre-COVID epoch. Patient access of healthcare also differed between the 2 time periods. Newly diagnosed patients in the during-COVID epoch were more likely to have been seen first virtually by a Pediatric Gastroenterologist as opposed to in-person (46.9% vs 0%, *P* < .01). Patients also exhibited symptoms for shorter duration prior to their first visit with a Gastroenterologist (5.99 months vs 4.53 months, *P* = .02). Patient diagnosed with IBD in the during-COVID epoch also utilized healthcare services more frequently in the 3 months following diagnosis including GI-specific appointments (1.48 visits vs 2.06 visits, *P* = 0.002) and email messages to their gastroenterologist (0.39 messages vs 1.36 messages, *P* = .006).

**Table 4.** New diagnosis specific participant clinical and healthcare utilization data.

		Pre-COVID (*N* = 72)	During-COVID (*N* = 67)	*P*-value
Clinical characteristics				
- Mental health diagnosis^a^	None	88.4% (61)	73.8% (48)	<.01*
	Depression	0	10.8% (7)	
	Anxiety	4.2% (3)	13.8% (9)	
	Both	7.2% (5)	1.5% (1)	
- Underlying atopy/autoimmune disorder^a^		21.5% (14)	18.6% (11)	.67
Pre-GI referral				
- Average duration of symptoms prior to GI visit^b^		5.99 months	4.53 months	.02*
- Time from PCP referral to GI visit^b^		27.71 days	28.33 days	.47
- Time from GI visit to diagnosis^b^		32.73 days	28.94 days	.36
Post-GI referral healthcare utilization				
- Type of initial GI visit^a^	In-person	100% (70)	53.1% (34)	<.01*
	Virtual	0	46.9% (30)	
- Location of endoscopy^a^	Ambulatory	81.4% (57)	83.3% (55)	.77
	Inpatient	18.6% (13)	16.7% (11)	
- Average number of utilized services 3 months post diagnosis^b^	Admissions	0.11 (0.36)	0.20 (0.51)	.12
	ED visits	0.17 (0.42)	0.33 (0.76)	.07
	Clinic appt	1.48 (0.90)	2.06 (1.28)	.002*
	MyChart	0.39 (1.16)	1.36 (2.77)	.006*
	Telephone calls	0.72 (1.17)	0.75 (1.60)	.46

Abbreviations: ED, emergency department; GI, gastroenterology; PCP, primary care physician.

^a^Statistical analysis for this variable performed with chi-squared test.

^b^Statistical analysis for this variable performed with independent *t*-test.

*Denotes statistical significance at α ≤ .05.

## Discussion

In this retrospective cohort study, there were similar numbers of new diagnoses of pediatric IBD and admissions for pediatric IBD flares between the pre-COVID and during-COVID epochs. However, the demographics of the participants and the severity of disease upon presentation for care differed significantly between the 2 epochs. Most notably, participants in the COVID-19 epoch exhibited more severe markers of disease, particularly elevated fecal calprotectin in CD patients and reductions in albumin in VEO-IBD patients. Our VEO-IBD group consisted of a small sample of patients and those findings need to be validated in a larger cohort. Patients in the during-COVID epoch were also more likely to be initiated on biologic therapies, suggesting more severe disease as perceived by patients’ pediatric gastroenterologist; however, that practice change might have been multifactorial and a result of limitations to telehealth versus in-person assessment. The demographics of the epochs varied in that participants during COVID were more likely to be non-Hispanic and living in urban areas as defined as population >50 000.^31^ Hispanic communities faced higher rates of COIVD-19 morbidity compared to other populations. However, the rates of case fatality among Hispanics are similar to those of other groups. This could be a result of disparities in healthcare access and increased exposure risk both of which could have influenced presented results.^32^ Factors contributing to the observed difference in disease severity between the 2 epochs are likely multifactorial as the COVID-19 pandemic has dramatically and differentially altered patients biological and social environments. Particularly, we hypothesize that changes in microbial environment and exposures, increases in the incidence of underlying mental health disorders, and changes in the delivery of care largely underlie the increased disease severity observed during the COVID-19 pandemic.

Microbial exposures can be impacted by behaviors such as mask wearing or from daily activities and it is possible that patients living in urban versus rural areas experienced differing degrees of social isolation during the COVID-19 pandemic and thus differential reductions in microbial exposures. Studies have reported that people in rural areas were more likely to continue communing in public places during COVID given the relative lack of access.^33–36^ It is possible that those experiencing more social isolation had a higher propensity for autoimmune/autoinflammatory activity per the hygiene hypothesis.^8,37^

For newly diagnosed patients with IBD, a greater percentage in the during-COVID epoch had preexisting depression and anxiety as compared to the pre-COVID epoch. This observation is consistent with the global rise in anxiety and depression across all pediatric patients during COVID.^38^ While anxiety and depression have not been identified as independent predictors or risk factors for IBD development, these disorders are correlated with worsened disease course, higher likelihood of hospital admissions and ED visits, thus could explain the increased disease severity seen in our during-COVID population.^39^ Research suggests that both individual and group-based cognitive behavioral therapy have demonstrated positive outcomes in reducing rates of anxiety and depression among certain individuals with IBD, possibly affecting the disease course.^40^ These results underscore the importance of depression/anxiety screening and therapy among patients with IBD and importance of mental health providers being involved in care of patients with IBD from diagnosis. We did not perform specific analysis assessing severity of disease in the patients with comorbid psychiatric disorder given our relatively small sample size and lack of power.

In addition, there were significant changes in the way care was delivered and how patients utilized healthcare services during COVID. Nearly half of patients during COVID were seen first via telemedicine by one of our pediatric gastroenterologists, in line with hospital-wide policy changes to prevent unnecessary exposures. This pivot to telemedicine also made patient email communication mainstream and likely accounts for the increased use of email messaging in the 3 months following their diagnosis. The telemedicine platform also resulted in new considerations for patient care. Ease of use and intuitiveness of the telemedicine platform along with patients having access to technology and internet could be limiting factors in patient adoption of telemedicine and therefore access to pediatric GI care at our institution. These barriers along with fear of exposure could have led to delayed access compared to the pre-COVID epoch and thus accounting for the more severe disease on presentation. However, in analysis of our study cohort, this change in practice to telemedicine did not lead to delays in scheduling patients’ initial GI visit or delays in the time between initial GI visit and diagnosis. This suggests our physicians were still able to discern which patients merited urgent endoscopy during virtual visits and were able to schedule needed endoscopic procedures in a timely manner. Furthermore, patient-reported average duration of symptoms prior to GI visit was actually shorter during COVID, suggesting patients did not choose to delay presentation to care during COVID. It is reassuring that patients received care in a timely manner during COVID despite challenges with scheduling, the transition to largely virtual services, and implementation of pre-procedure testing policies.^41^ Of note, while not significant, patients in the during-COVID epoch were more likely to be seen in the ED or admitted to the hospital, which could reflect patient hesitance to seek medical care during the pandemic with uncertainty over the epidemiology COVID or potentially from healthcare access challenges.

We have not specifically assessed social determinants of health in our sample. Elements of those, like policies, programs, and interventions aimed at reducing socioeconomic inequalities, improving education, creating healthy environments, promoting social support networks, and ensuring equitable access to healthcare were likely all affected during the COVID pandemic.

Overall, the time-matched nature of this review allows comparison of before and during COVID times, but also controls for any impact that seasonality may have on diagnosis and disease severity. Our tertiary care center is unique in that it serves the entirety of the state’s pediatric population, meaning a large portion of our patient population lives in rural areas. This study is limited in part by its design; only able to identify correlation and not assert causation. Furthermore, retrospective chart reviews are susceptible to documentation errors and variation in electronic health record charting methods. Given this sample is taken from 1 tertiary care center, its size is limited and less generalizable to other sites that serve a primarily urban population.

In conclusion, this retrospective chart review identified increased disease severity in newly diagnosed pediatric patients with IBD as well as pediatrics patients admitted for flare, despite stable rates of admission and diagnosis. Given more patients during COVID lived in rural areas where microbial isolation was likely less pronounced suggests a role of the hygiene hypothesis in underlying increases in inflammation susceptibility and worsening of disease severity. Dysbiosis due to drop in microbiome diversity related to social isolation, lack of microbial exposures, and westernized lifestyle have long been suspected in IBD etiology.^42–44^ Furthermore, increases in anxiety and depression rates during COVID may have also contributed to worsened disease severity. Hospital-wide practice changes including increased utilization of virtual visits did not seem to delay diagnosis and patients utilized their online health portals more often to get in contact with their pediatric gastroenterologists. In our practice setting, access to care without telemedicine would have been very limited. Further study of the differences in microbial exposures across rural and urban households including the study of individual patient microbiomes is an important next step in elucidating the role of the hygiene hypothesis in disease progression and severity.

## Data Availability

Data from this study is not publicly available.
